# Prevalence and genetic diversity of *Babesia microti* in rodents from central and southern Shanxi, China

**DOI:** 10.1186/s13071-025-06898-6

**Published:** 2025-06-22

**Authors:** Fei Ren, Yiping Liu, Jingrong Niu, Yang Song, Hongbing Cheng, Chao Zhao, Jia Cui, Yunxia Chen, Yuzan Bai, Huaxiang Rao, Juan Yu

**Affiliations:** 1 Department of Laboratory Animal Center, Changzhi Medical College, Changzhi, 046000 China; 2 Department of Basic Medical Sciences, Changzhi Medical College, Changzhi, 046000 China; 3 Department of Public Health and Preventive Medicine, Changzhi Medical College, Changzhi, 046000 China; 4 Shanxi Higher Education Institutions of Science and Technology Innovation Plan Platform, Laboratory of Environmental Factors and Population Health, Changzhi, 046000 China; 5 Key Laboratory of Environmental Pathogenic Mechanisms and Prevention of Chronic Diseases, Changzhi Medical College, Changzhi, 046000 China

**Keywords:** *Babesia microti*, Kobe type, Rodents, Genetic diversity

## Abstract

**Background:**

Babesiosis, a globally emerging tick-borne zoonosis caused by intraerythrocytic protozoan *Babesia* species, poses a significant threat to both animal and human health. This study investigated the prevalence and genetic diversity of *Babesia* sp. in small rodents in central and southern Shanxi Province, China.

**Methods:**

Rodents were captured from central and southern Shanxi Province, China. Liver, spleen, and kidney specimens were collected and screened for *Babesia* sp. based on *18S rRNA* gene amplification and sequencing. For genetic and evolutionary analysis of *Babesia* sp. sequences based on the *18S rRNA* gene, a phylogenetic tree was created using MEGA 11. Genetic diversity was analyzed using DNASP 6.12.03, and haplotype networks in *Babesia microti* from different regions and hosts were constructed using PopART software.

**Results:**

Three hundred and one rodents were captured; PCR screening revealed a 6.64% (20/301) prevalence of *Babesia* sp. infection, detected in *Niviventer confucianus* (16.87%, 14/83) and *Apodemus agrarius* (3.85%, 6/156). Detection rates did not differ significantly according to sex, tissue, or habitat type. Geographically, central Shanxi exhibited significantly higher detection rates than southern Shanxi (9.74% vs. 0.94%, *χ*^2^ = 8.573, *P* = 0.003). Phylogenetic analysis of the partial *18S rRNA* gene (1083 bp) confirmed that all sequences obtained in this study were the *B. microti* Kobe type, closely related to sequences from southeastern Shanxi obtained in our previous study (with 99.7–100% identity), with the ability to infect humans. Genetic diversity analysis of 65 *B. microti* sequences from China (20 sequences from the present study and 45 from GenBank) identified 21 haplotypes with host- and geography-specific patterns. Host-specific analysis of *18S rRNA* gene polymorphisms revealed higher genetic diversity in tick-derived sequences than in rodent- or human-derived sequences. Haplotype network analysis suggested that Shanxi sequences (Hap-1, Hap-10, and Hap-11) exhibited close genetic proximity of 1–3 nucleotide substitutions with rodent-derived sequences from Yunnan and Fujian provinces and human-derived sequences from Yunnan and Zhejiang provinces.

**Conclusions:**

This study found a high prevalence and low genetic diversity of *B. microti* infection in wild rodents in central Shanxi, which could provide a basis for local corresponding prevention and control strategies.

**Graphical Abstract:**

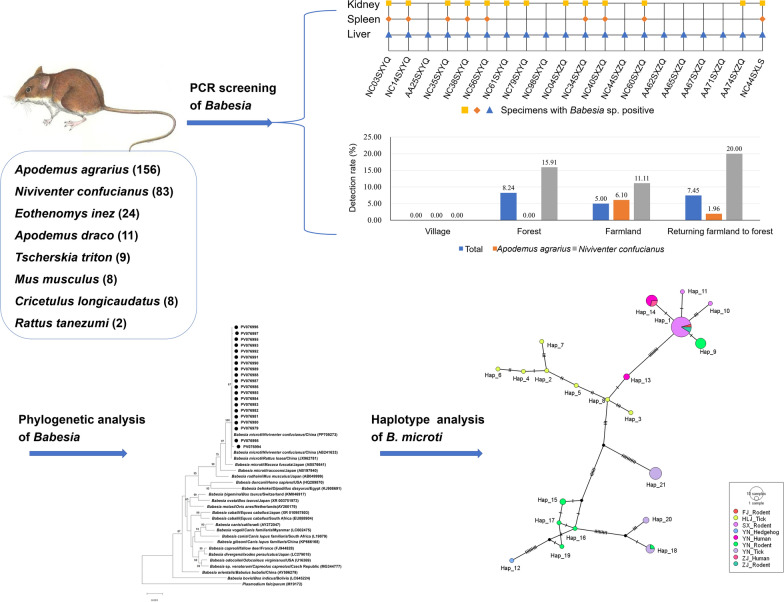

**Supplementary Information:**

The online version contains supplementary material available at 10.1186/s13071-025-06898-6.

## Background

Babesiosis is an emerging infectious disease caused by *Babesia* species of intraerythrocytic, tick-borne protozoan parasites, belonging to the suborder Piroplasmidea and family Babesiidae [1, 2]. Over 120 *Babesia* species have been identified worldwide, with *Babesia microti*, *B. venatorum*, *B. duncani*, *B. divergens*, and *B. crassa*-like recognized as primary human pathogens [3, 4]. *Babesia* sp. can cause mild-to-severe infection in humans, which is generally asymptomatic or mild in healthy people; however, the high morbidity and mortality in immunodeficient and older patients show clinical features similar to those of malaria [5]. Cases of human babesiosis have been reported in Europe, Asia, Africa, Australia, and South America [2]. As the number of human cases increases, babesiosis is becoming a significant threat to public health worldwide [6].

*Babesia* sp. exhibits remarkable host adaptability, infecting a broad spectrum of mammals including rodents, cervids, and domestic animals [7–12]. Rodents, particularly wild rodent populations such as *Niviventer confucianus*, *Apodemus agrarius* [13], *A. chevrieri*, *N. fulvescens* [14], *A. speciosus*, *Clethrionomys rufocanus* [15], *Peromyscus leucopus* [15], *Microtus arvalis*, *M. agrestis*, and *M. oeconomus* [16, 17], serve as critical reservoir hosts and play important roles in the conservation, transmission, and prevalence of babesiosis [18].

Our previous study showed that *B. microti* was found in wild rodents in southeastern Shanxi [19]. In this study, we aimed to investigate the presence of *Babesia* sp. in rodents from central and southern Shanxi to provide a more comprehensive understanding of the prevalence of *Babesia* sp. in Shanxi Province and to take corresponding local prevention and control measures.

## Methods

### Study sites and rodent collection

From July 8 to July 29, 2024, rodent specimens were collected using the night-trapping method [22] in the village, farmland, forest, and RFF (an acronym for returning farmland to forest) of Chakou Township (Pingding County, Yangquan City, located in central Shanxi), Xinanyu Township (Suburban District, Yangquan City, located in central Shanxi), Matian Town, Shixia Township, and Tongyu Town (Zuoquan County, Jinzhong City, located in central Shanxi), and Lishan Town and Gucheng Town (Yuanqu County, Yuncheng City, located in southern Shanxi). Approximately 500 rodent traps baited with peanuts were deployed daily at 8:00 p.m., positioned at 5-m intervals, and retrieved by 5:00 AM the following morning. Rodents were killeded on site immediately after capture and subsequently transported to the laboratory for further dissection within a biosafety cabinet. For anesthesia, the captured rodents were placed in a transparent plastic box with degreased cotton soaked in isoflurane. Euthanasia was performed by cervical dislocation under deep anesthesia, which resulted in efficient and quick death and minimized pain. After death, rodents’ liver, kidney, and spleen tissues were collected and stored at – 80 ℃ for later use.

A total of 301 small rodents were captured and classified into eight species based on their morphology and mitochondrial cytochrome C oxidase subunit I (CO I) gene [23]: *Apodemus agrarius* (*n* = 156), *Niviventer confucianus* (*n* = 83), *Eothenomys inez* (*n* = 24), *Apodemus draco* (*n* = 11), *Tscherskia triton* (*n* = 9), *Mus musculus* (*n* = 8), *Cricetulus longicaudatus* (*n* = 8), and *Rattus tanezumi* (*n* = 2) (Table 1).

**Table 1:** Information on rodent samples

Sampling sites	Habitats	Host								No. captured
		AA	NC	EI	AD	TT	MM	CL	RT	
Site 1-Chakou	Forest	1	1	0	0	0	0	0	0	2
	RFF	11	1	0	0	0	0	0	0	12
Site 2-Xinanyu	Forest	4	5	0	0	0	0	0	0	9
	Farmland	2	0	0	0	0	3	0	0	5
	RFF	40	29	0	0	7	1	5	0	82
Site 3-Matian	Village	0	0	0	0	0	0	0	2	2
	Forest	7	19	1	2	0	2	0	0	31
	Farmland	24	1	2	0	0	1	0	0	28
Site 4-Shixia	Forest	2	15	0	1	0	0	0	0	18
Site 5-Tongyu	Farmland	6	0	0	0	0	0	0	0	6
Site 6-Lishan	Forest	9	4	6	3	1	0	2	0	25
	Farmland	14	4	15	5	1	0	1	0	40
Site 7-Gucheng	Farmland	36	4	0	0	0	1	0	0	41
Total		156	83	24	11	9	8	8	2	301

AA: *Apodemus agrarius*, NC: *Niviventer confucianus*, EI: *Eothenomys inez*, AD: *Apodemus draco*, TT: *Tscherskia triton*, MM: *Mus musculus*, CL: *Cricetulus longicaudatus*, RT: *Rattus tanezumi*

RFF: returning farmland to forest

### *Babesia* molecular detection

DNA was extracted from approximately 10 mg of liver, kidney, and spleen tissues using a TIANamp Micro DNA Kit [TIANGEN Biotech (Beijing) Co., Ltd., China] according to the manufacturer’s protocols. The partial *18S rRNA* gene (488 bp) amplification was performed in 20-μl mixtures containing 10 μl 2 × EasyTaq® PCR SuperMix (TransGen Biotech, China), 6.4 μl double-distilled H_2_O, 0.8 μl (10 μmol/l) of each primer (forward: 5ʹ-GTCTTGTAATTGGAATGATGG-3ʹ, reverse: 5ʹ-TAGTTTATGGTTAGGACTACG-3ʹ) [24], and 2 μl of DNA template. The amplification was performed under the following conditions: one cycle for 10 min at 94 °C; 35 cycles for 30 s at 94 °C, 30 s at 55 °C, and 45 s at 72 °C; and a final extension for 10 min at 72 °C. The PCR products were identified by 1.5% agarose gel electrophoresis.

For *Babesia* sp.-positive specimens, a partial *18S rRNA* sequence (1198 bp) of *B. microti* was amplified using nested PCR [14], outer primers 155 F 5′- CTAGGGCTAATACATGCTCG-3′ and 1606R 5′- ACTAGGCATTCCTCGTTC-3′ for the first PCR amplification and inner primers 255 F 5′-AAATTAGCGAATCGCATGG-3′ and 1453R 5′-ACAGACCTGTTATTGCCTTAC-3′ for the second PCR amplification. Primary and nested PCR amplifications were performed in 20- and 50-μl mixtures, respectively, according to the reaction described above, and 1 μl of the first-round PCR product was used for the second amplification. The conditions for both amplification rounds were 94 °C for 5 min; 35 cycles of 94 °C for 30 s, 53 °C for 40 s and 72 °C for 90 s; followed by a final extension step at 72 °C for 7 min. Next, 10-μl PCR products were identified by 1.5% agarose gel electrophoresis, and then the remaining PCR products were sent to Sangon Biotech (Shanghai, China) for sequencing.

### Sequencing and phylogenetic analysis

Sequences generated in this study were submitted to GenBank (accession nos. PV076979-PV076998). The obtained sequences were compared using BLAST against related *Babesia* species sequences in GenBank (http://blast.ncbi.nlm.nih.gov/ Blast.cgi). Additionally, related *18S rRNA* gene sequences of *Babesia* species were downloaded from GenBank and used to construct a phylogenetic tree. The phylogenetic trees were created by using neighbor-joining (NJ), maximum likelihood (ML), and minimum-evolution (ME) methods in MEGA version 11, respectively [25]. *Plasmodium falciparum* was used as the outgroup in *Babesia* identification, and *Babesia rodhaini* was used as the outgroup in *B. microti* identification.

### Genetic diversity analysis

As of February 5, 2025, there were 64 *18S rRNA* gene sequences of *B. microti* from China have been deposited in GenBank. Of these, 45 sequences > 1000 bp in length with clear origins (including location and host information) were selected for genetic polymorphism analysis. *Babesia microti* sequences were analyzed for polymorphisms based on the number of polymorphic sites (S), the number of haplotypes (H), nucleotide diversity (π), average number of nucleotide differences (κ), and haplotype diversity (Hd) using DNASP 6.12.03. Sequences were analyzed based on a TCS network using Population Analysis with Reticulate Trees (PopART) software, version 1.7 (http://popart.otago.ac.nz/index. shtml).

### Statistical analysis

The detection rates of *Babesia* sp. in different small rodent sexes and tissues and different sampling sites and habitats were analyzed using the chi-square test. Then, multivariate logistic regression analysis was performed on variables showing statistical significance in univariate analyses to explore risk factors associated with *Babesia* sp. occurrence in rodents (comparing *Babesia* DNA-positive vs. DNA-negative samples). Odds ratios (ORs) with 95% confidence intervals (CIs) were calculated to quantify association strength. All data were analyzed using SPSS 22.0 (SPSS, Inc., Chicago, IL, USA). *P* < 0.05 was considered statistically significant.

## Results

### *Babesia* infections

**Fig. 1:**
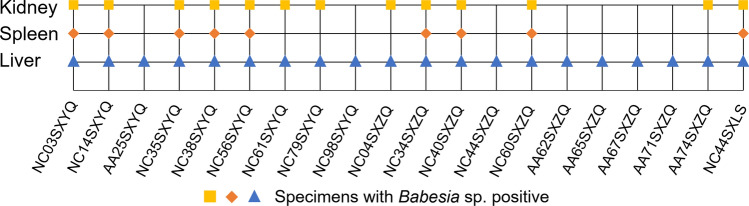
Detection of *Babesia* species in various tissues of naturally infected rodents

*Babesia* sp. was considered positive when it was detected in any tissue of rodents using PCR. *Babesia* sp. was detected in the liver, kidney, and spleen of nine rodents, the liver and kidney of four rodents, and the liver of seven rodents (Fig. 1). In total, 20 rodents tested positive for *Babesia* sp. infection, with an infection rate of 6.64% (20/301). The detection rates of *Babesia* sp. were 6.64% (20/301) in the liver, 3.00% (9/300) in the spleen, and 4.32% (13/301) in the kidney, respectively, and the differences were not statistically significant (*χ*^2^ = 4.611, *P* = 0.100) (Table 2). The detection rates were 6.58% (10/152) in females and 6.71% (10/149) in males, which were not statistically significant (*χ*^2^ = 0.002, *P* = 0.963). Among eight captured rodent species, *Babesia* sp. was exclusively detected in two rodent species: *N*. *confucianus*, with a detection rate of 16.87% (14/83), and *A*. *agrarius*, with a detection rate of 3.85% (6/156), and the difference in the detection rate among rodent species was statistically significant (*χ*^2^ = 11.979, *P* < 0.001) (Table 2).

**Table 2:** Detection rate of *Babesia* infection in different tissues of small rodents

Host	No. detection	No. PCR positive in liver (%)	No. PCR positive in spleen (%)	No. PCR positive in kidney (%)	Total (%)
AA	156	6(3.85)	0(0.00)	1(0.64)	6(3.85)
NC	83	14(16.87)	9(10.84)	12(14.46)	14(16.87)
EI	24	0(0.00)	0(0.00)	0(0.00)	0(0.00)
AD	11	0(0.00)	0(0.00)	0(0.00)	0(0.00)
TT	9	0(0.00)	0(0.00)	0(0.00)	0(0.00)
MM	8	0(0.00)	0(0.00)	0(0.00)	0(0.00)
CL	8	0(0.00)	0(0.00)	0(0.00)	0(0.00)
RT	2	0(0.00)	0(0.00)	0(0.00)	0(0.00)
Total	301	20(6.64)	9 (3.00)	13(4.32)	20(6.64)

AA: *Apodemus agrarius*, NC: *Niviventer confucianus*, EI: *Eothenomys inez*, AD: *Apodemus draco*, TT: *Tscherskia triton*, MM: *Mus musculus*, CL: *Cricetulus longicaudatus*, RT: *Rattus tanezumi*

One spleen-deficient specimen of *Apodemus agrarius* with serial number AA74SXLS, collected from Gucheng Town, Yuanqu County, Yuncheng City

*Babesia* sp. was detected in rodents from the forest, farmland, and RFF but not in the village. The highest detection rate was observed in forests (8.24%), followed by RFF (7.45%) and farmland (5.00%). However, no statistically significant differences were observed among the three habitats (*χ*^2^ = 0.961, *P* = 0.619) (Table 3). Among the seven sampling sites, *Babesia* sp. was detected in rodents from five townships, excluding Site 4-Shixia (forest, Jinzhong) and Site 7-Gucheng (farmland, Yuncheng). The highest detection rate occurred in Site 5-Tongyu (farmland, Jinzhong) at 33.33%, followed by Site 3-Matian (RFF, Jinzhong) at 13.11% (Table 4). A significantly higher *Babesia* sp. detection rate was observed in central Shanxi (Yangquan and Jinzhong: 9.74%, 19/195) than in southern Shanxi (Yuncheng: 0.94%, 1/106) (*χ*^2^ = 8.573, *P* = 0.003) (Table 4).

**Table 3:** Detection rate of *Babesia* infection of small rodents in different habitats

Habitats	Host								Detection rate (%)
	AA	NC	EI	AD	TT	MM	CL	RT	
Village	0	0	0	0	0	0	0	2	0/2(0.00)
Forest	23	44	7	6	1	2	2	0	7/85(8.24)
Farmland	82	9	17	5	1	5	1	0	6/120(5.00)
RFF	51	30	0	0	7	1	5	0	7/94(7.45)
Total	156	83	24	11	9	8	8	2	20/301(6.64)

AA: *Apodemus agrarius*, NC: *Niviventer confucianus*, EI: *Eothenomys inez*, AD: *Apodemus draco*, TT: *Tscherskia triton*, MM: *Mus musculus*, CL: *Cricetulus longicaudatus*, RT: *Rattus tanezumi*

RFF: returning farmland to forest

**Table 4:** Detection rate of *Babesia* infection of small rodents in different sampling sites

Sampling sites	Host								Detection rate (%)
	AA	NC	EI	AD	TT	MM	CL	RT	
Chakou	12	2	0	0	0	0	0	0	1/14(7.14)
Xinanyu	46	34	0	0	7	4	5	0	8/96(8.33)
Matian	31	20	3	2	0	3	0	2	8/61(13.11)
Shixia	2	15	0	1	0	0	0	0	0/18(0.00)
Tongyu	6	0	0	0	0	0	0	0	2/6(33.33)
Lishan	23	8	21	8	2	0	3	0	1/65(1.54)
Gucheng	36	4	0	0	0	1	0	0	0/41(0.00)
Total	156	83	24	11	9	8	8	2	20/301(6.64)

AA: *Apodemus agrarius*, NC: *Niviventer confucianus*, EI: *Eothenomys inez*, AD: *Apodemus draco*, TT: *Tscherskia triton*, MM: *Mus musculus*, CL: *Cricetulus longicaudatus*, RT: *Rattus tanezumi*

To determine risk factors associated with *Babesia* sp. occurrence in rodents, we then performed multivariate logistic regression analysis. As only *A. agrarius* and *N. confucianus* tested positive for *Babesia* sp. in this study, the multivariate analysis was restricted to these two rodent species, with geographic region and rodent species included as variables. The analysis indicated that the infection risk in central Shanxi was higher than in southern Shanxi (*OR* = 6.20; 95% *CI*: 0.793–48.463; *P* > 0.05), although this difference was not statistically significant. In contrast, *N*. *confucianus* had a significantly higher infection risk compared to *A*. *agrarius* (*OR* = 3.97; 95% *CI*: 1.442–10.950; *P* < 0.01) (Table 5).

**Table 5:** Multivariate logistic regression analysis of *Babesia* sp. infection risk

Factor		No. captured	No. PCR positive	Detection rate (95% CI, %)	*b*	SE	*P*-value	*OR* (95% *CI*)
Geographic region	Southern Shanxi	71	1	1.41 (− 1.40, 4.22)	–	–	–	–
	Central Shanxi	168	19	11.31 (6.47, 16.15)	1.825	1.049	0.082	6.201 (0.793, 48.463)
Rodent species	*A*. *agrarius*	156	6	3.85 (0.79, 6.90)	–	–	–	–
	*N*. *confucianus*	83	14	16.87 (8.64, 25.09)	1.380	0.517	0.008	3.973 (1.442, 10.950)

*CI* confidence interval, *OR* odds ratio

Reference group: southern Shanxi, *Apodemus* *agrarius*

### Identification of *Babesia microti*

BLAST analyses demonstrated that the 20 *18S rRNA* gene sequences (1083 bp, after removing low-quality bases at both ends of the sequence) were *B. microti*, including 14 sequences from *N*. *confucianus* and six sequences from *A*. *agrarius*.

**Fig. 2:**
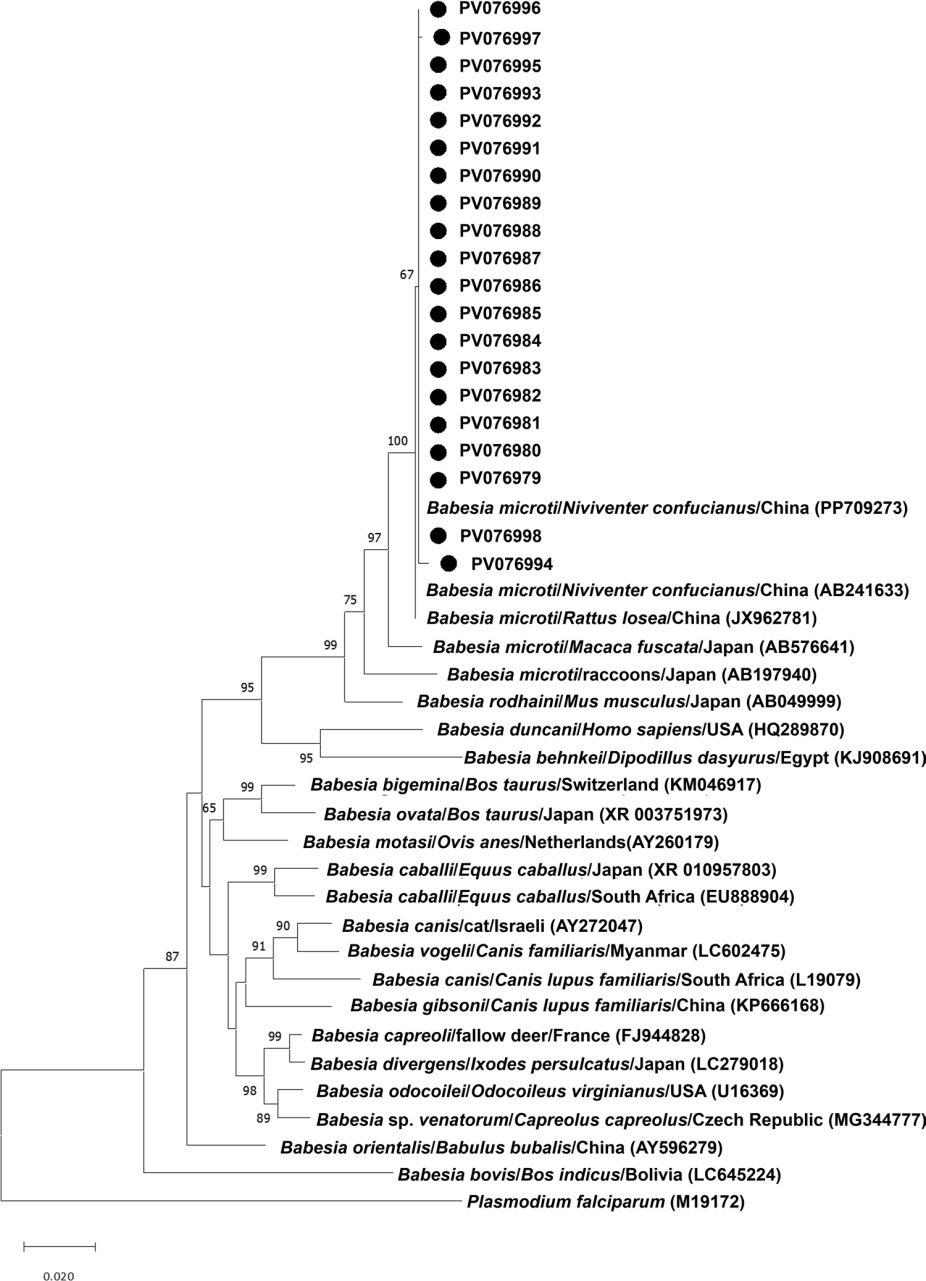
Neighbor-joining phylogenetic tree of *18S rRNA* gene of *Babesia* species. The tree was constructed by using the neighbor-joining (NJ) method with the Kimura 2-parameter model, bootstrap values calculated with 1000 replicates. Sequences obtained in this study are indicated by black dots. The *Babesia* species, host, region, and accession number are given

We constructed phylogenetic trees using NJ, ML, and ME methods, respectively. For phylogenetic analysis of *Babesia* sp. based on *18S rRNA* gene sequences, all three methods yielded similar results. Our sequences formed a robust cluster with *B. microti* references and demonstrated closest genetic affinity (99.7–100% identity) to the *B. microti* sequence derived from *N. confucianus* in our previous study (accession PP709273) (Fig. 2 and Figure S1).

**Fig. 3:**
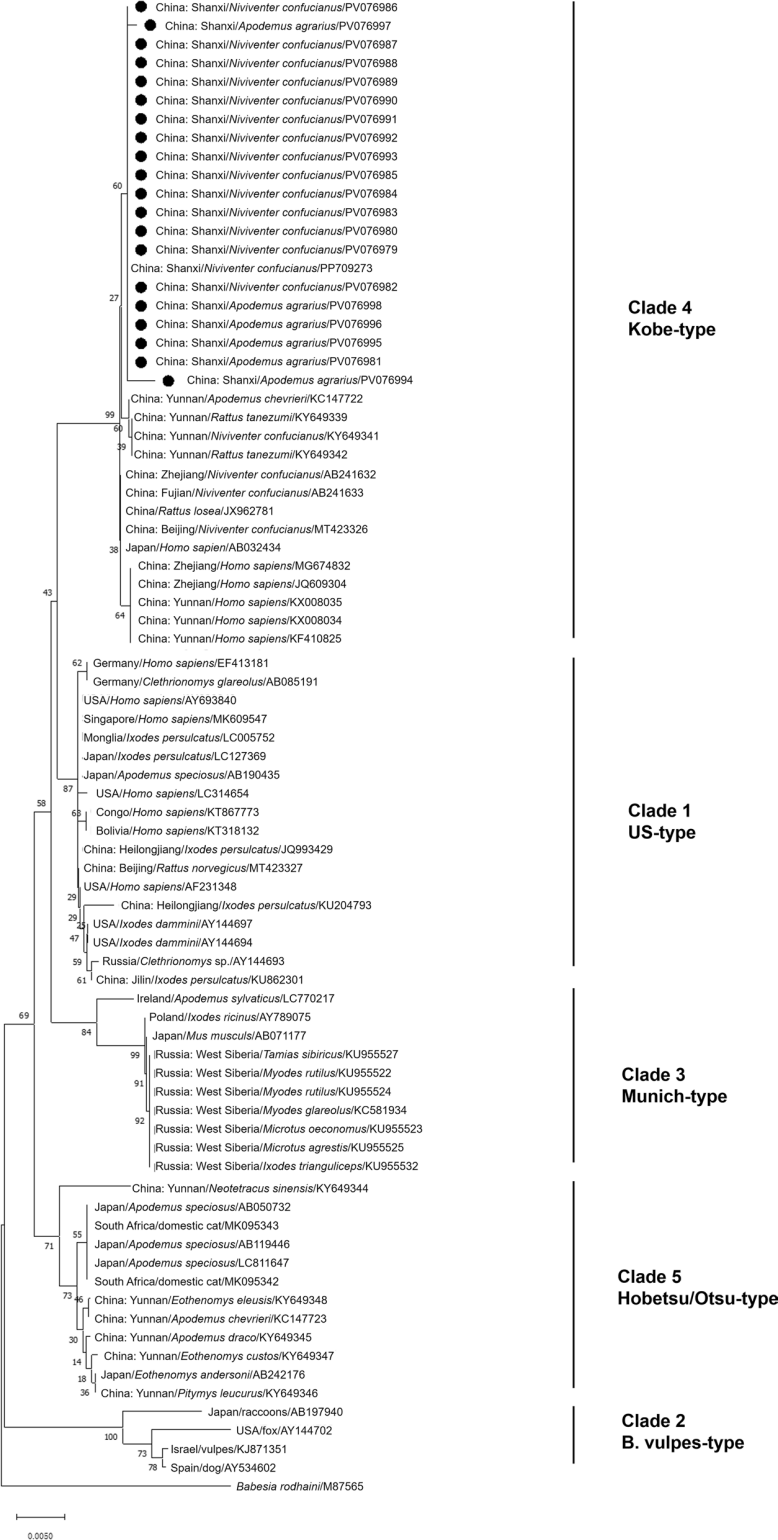
Neighbor-joining phylogenetic tree of *18S rRNA* gene of *Babesia microti*. The tree was constructed by using the neighbor-joining (NJ) method with the Kimura 2-parameter model, bootstrap values calculated with 1000 replicates. Sequences obtained in this study are indicated by black dots. The host, region, and accession number are given

Divergent phylogenetic patterns emerged when specifically analyzing *B. microti 18S rRNA* sequences. The ML-generated tree exhibited methodological limitations, as evidenced by the failure of the designated outgroup (*B. rodhaini*) to form a distinct cluster, indicating ML’s unsuitability for this particular analysis. In contrast, both NJ and ME methods produced phylogenetically consistent results, revealing five well-supported clades (Clades 1–5) within *B. microti*. This clade structure aligns with established taxonomic classifications [26, 27], with the Shanxi *B. microti* sequences unequivocally grouping within Clade 4 Kobe type (Fig. 3 and Figure S2).

### Genetic diversity analysis of *Babesia microti*

Analysis of *18S rRNA* gene polymorphisms (1056 bp) across 65 sequences of *B. microti* (20 sequences from this study and 45 from GenBank) identified 48 polymorphic loci (S = 48) and 21 haplotypes (H = 21); Hap-1, Hap-10, and Hap-11 were observed in our study. Haplotype diversity (Hd = 0.857 ± 0.034), average nucleotide difference (κ = 10.318), and nucleotide diversity (π = 0.01008) were observed.

**Fig. 4:**
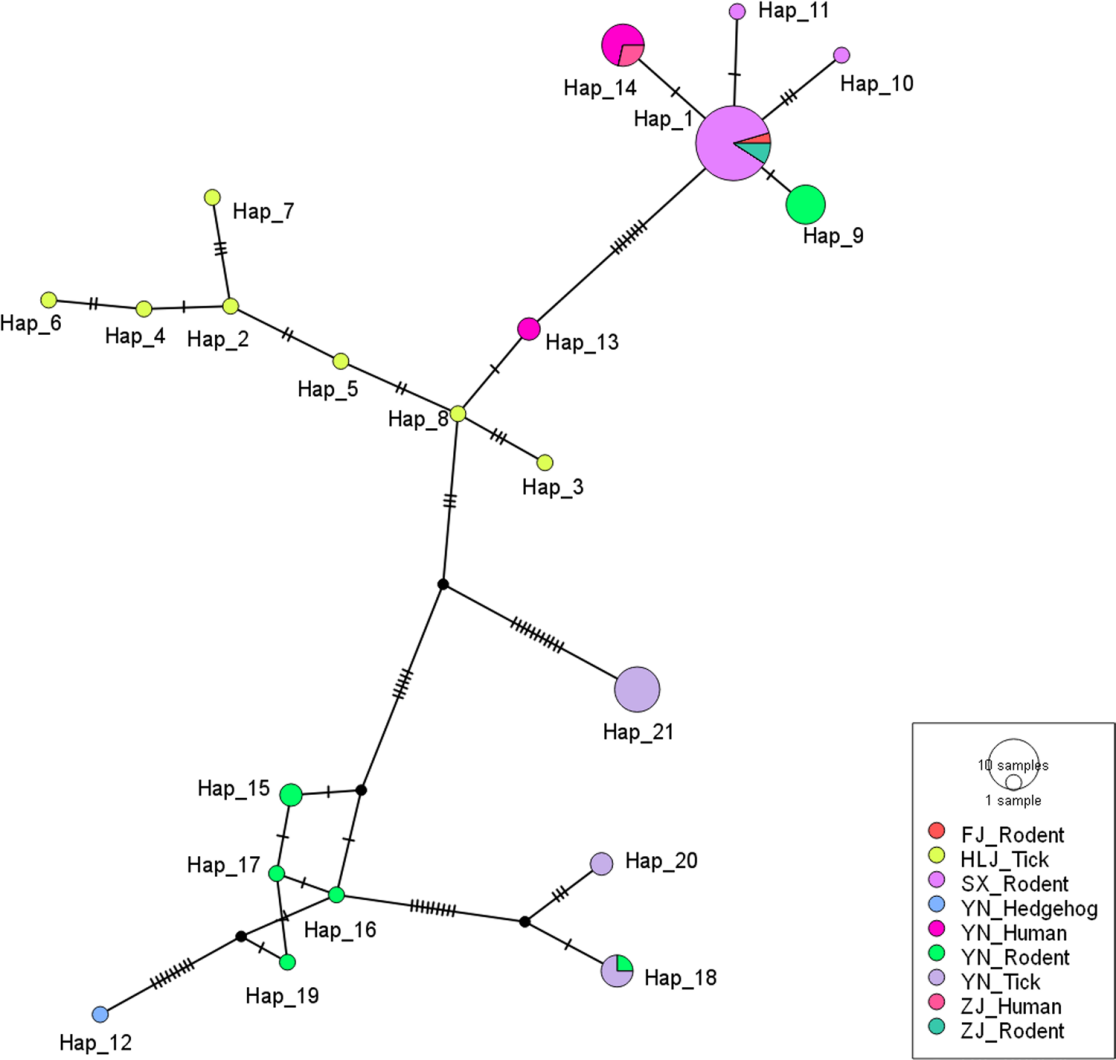
Haplotype networks of *18S rRNA* gene in *Babesia microti* from different regions and hosts. In the haplotype network, a circle represents a haplotype, and the line connecting two circles indicates that these two haplotypes are related (one is a mutation of the other). The short vertical lines on the line represent the number of base substitutions required to go from one haplotype to the connected one, with each vertical line representing one substitution. The colored circles represent the haplotypes actually sampled, and the size of the circle indicates the number of such haplotypes. The gray circles represent the inferred intermediate haplotypes that may exist but were not sampled. FJ: Fujian Province, HLJ: Heilongjiang Province, SX: Shanxi Province, YN: Yunnan Province, ZJ: Zhejiang Province

Based on the current *18S rRNA* sequences observed in China, *B. microti* exhibits 21 haplotypes with distinct host and geographic specificity. The Heilongjiang sequences derived from ticks were Hap-2 to Hap-8. The Zhejiang sequences derived from humans were Hap-14, and the Zhejiang and Fujian sequences derived from rodents were Hap-1. Yunnan displayed the most diverse sources: human-derived sequences corresponding to Hap-13 and Hap-14, rodent sequences corresponding to Hap-9 and Hap-15 ~ 19, and tick sequences corresponding to Hap-18, Hap-20, and Hap-21; the hedgehog sequence was identified as Hap-12. All Shanxi sequences were exclusively sourced from rodents, comprising Hap-1, Hap-10, and Hap-11, and exhibited close genetic proximity by 1–3 nucleotide substitutions with rodent-derived sequences from Yunnan and Fujian provinces and human-derived sequences from Yunnan and Zhejiang provinces (Table 6, Fig. 4).

**Table 6:** Haplotypes of *Babesia microti* sequences in China

Haplotype	Acc. no.	Host	Species	Site
Hap-1	AB241633	Rodent	*Niviventer confucianus*	China: Fujian
Hap-1	**PV076995**	**Rodent**	***Apodemus agrarius***	**China: Shanxi**
Hap-1	**PV076996**	**Rodent**	***Apodemus agrarius***	**China: Shanxi**
Hap-1	**PV076998**	**Rodent**	***Apodemus agrarius***	**China: Shanxi**
Hap-1	**PV076981**	**Rodent**	***Apodemus agrarius***	**China: Shanxi**
Hap-1	PP709273	Rodent	*Niviventer confucianus*	China: Shanxi
Hap-1	**PV076979**	**Rodent**	***Niviventer confucianus***	**China: Shanxi**
Hap-1	**PV076980**	**Rodent**	***Niviventer confucianus***	**China: Shanxi**
Hap-1	**PV076982**	**Rodent**	***Niviventer confucianus***	**China: Shanxi**
Hap-1	**PV076983**	**Rodent**	***Niviventer confucianus***	**China: Shanxi**
Hap-1	**PV076984**	**Rodent**	***Niviventer confucianus***	**China: Shanxi**
Hap-1	**PV076985**	**Rodent**	***Niviventer confucianus***	**China: Shanxi**
Hap-1	**PV076986**	**Rodent**	***Niviventer confucianus***	**China: Shanxi**
Hap-1	**PV076987**	**Rodent**	***Niviventer confucianus***	**China: Shanxi**
Hap-1	**PV076988**	**Rodent**	***Niviventer confucianus***	**China: Shanxi**
Hap-1	**PV076989**	**Rodent**	***Niviventer confucianus***	**China: Shanxi**
Hap-1	**PV076990**	**Rodent**	***Niviventer confucianus***	**China: Shanxi**
Hap-1	**PV076991**	**Rodent**	***Niviventer confucianus***	**China: Shanxi**
Hap-1	**PV076992**	**Rodent**	***Niviventer confucianus***	**China: Shanxi**
Hap-1	**PV076993**	**Rodent**	***Niviventer confucianus***	**China: Shanxi**
Hap-1	AB241631	Rodent	*Niviventer confucianus*	China: Zhejiang
Hap-1	AB241632	Rodent	*Niviventer confucianus*	China: Zhejiang
Hap-2	KU204793	Tick	*Ixodes persulcatus*	China: Heilongjiang
Hap-3	KU204794	Tick	*Ixodes persulcatus*	China: Heilongjiang
Hap-4	KU204795	Tick	*Ixodes persulcatus*	China: Heilongjiang
Hap-5	KU204796	Tick	*Ixodes persulcatus*	China: Heilongjiang
Hap-6	KU204797	Tick	*Ixodes persulcatus*	China: Heilongjiang
Hap-7	KU204798	Tick	*Ixodes persulcatus*	China: Heilongjiang
Hap-8	JQ993429	Tick	*Ixodes persulcatus*	China: Heilongjiang
Hap-9	KY649343	Rodent	*Rattus tanezumi*	China: Yunnan
Hap-9	KC147722	Rodent	*Apodemus chevrieri*	China: Yunnan
Hap-9	KY649341	Rodent	*Niviventer confucianus*	China: Yunnan
Hap-9	KY649340	Rodent	*Rattus brunneusculus*	China: Yunnan
Hap-9	KY649339	Rodent	*Rattus tanezumi*	China: Yunnan
Hap-9	KY649342	Rodent	*Rattus tanezumi*	China: Yunnan
Hap-10	**PV076994**	**Rodent**	***Apodemus agrarius***	**China: Shanxi**
Hap-11	**PV076997**	**Rodent**	***Apodemus agrarius***	**China: Shanxi**
Hap-12	KY649343	Hedgehog	*Neotetracus sinensis*	China: Yunnan
Hap-13	KF410824	Human		China: Yunnan
Hap-13	KF410826	Human		China: Yunnan
Hap-14	KF410825	Human		China: Yunnan
Hap-14	KF410827	Human		China: Yunnan
Hap-14	KX008034	Human		China: Yunnan
Hap-14	KX008035	Human		China: Yunnan
Hap-14	KX008036	Human		China: Yunnan
Hap-14	JQ609304	Human		China: Zhejiang
Hap-14	MG674832	Human		China: Zhejiang
Hap-15	KC147723	Rodent	*Apodemus chevrieri*	China: Yunnan
Hap-15	KY649348	Rodent	*Eothenomys eleusis*	China: Yunnan
Hap-16	KY649345	Rodent	*Apodemus draco*	China: Yunnan
Hap-17	KY649347	Rodent	*Eothenomys custos*	China: Yunnan
Hap-18	KC147724	Rodent	*Mus pahari*	China: Yunnan
Hap-18	MH208601	Tick	*Ixodes* sp.	China: Yunnan
Hap-18	MH208602	Tick	*Ixodes* sp.	China: Yunnan
Hap-18	MH208608	Tick	*Ixodes* sp.	China: Yunnan
Hap-19	KY649346	Rodent	*Pitymys leucurus*	China: Yunnan
Hap-20	MH208603	Tick	*Ixodes* sp.	China: Yunnan
Hap-20	MH208611	Tick	*Ixodes* sp.	China: Yunnan
Hap-21	MH208604	Tick	*Ixodes* sp.	China: Yunnan
Hap-21	MH208605	Tick	*Ixodes* sp.	China: Yunnan
Hap-21	MH208606	Tick	*Ixodes* sp.	China: Yunnan
Hap-21	MH208607	Tick	*Ixodes* sp.	China: Yunnan
Hap-21	MH208609	Tick	*Ixodes* sp.	China: Yunnan
Hap-21	MH208610	Tick	*Ixodes* sp.	China: Yunnan
Hap-21	MH208612	Tick	*Ixodes* sp.	China: Yunnan

Sequences in this study are shown in bold

Host-specific analysis of *18S rRNA* polymorphism revealed higher genetic diversity in tick-derived sequences (Hd = 0.854 ± 0.069; 19 sequences; Hap-2 ~ 8, Hap-18, Hap-20 ~ 21) compared to rodent- (Hd = 0.608 ± 0.087; 36 sequences; Hap-1, Hap-9 ~ 11, Hap-15 ~ 19) and human-derived sequences (Hd = 0.389 ± 0.164; 9 sequences; Hap-13 ~ 14) (Table 7).

**Table 7:** DNA polymorphisms of *18S rRNA* gene in *Babesia microti* from different hosts

Hosts	n	S	H	κ	π	Hd (mean ± SD)	Haplotype (*n*)
Tick	19	35	10	12.877	0.01255	0.854 ± 0.069	Hap-2 (1), Hap-3 (1), Hap-4 (1), Hap-5 (1), Hap-6 (1), Hap-7 (1), Hap-8 (1), Hap-18 (3), Hap-20 (2), Hap-21 (7)
Rodent	36	28	9	5.430	0.00520	0.608 ± 0.087	Hap-1(22), Hap-9 (6), Hap-10 (1), Hap-11 (1), Hap-15 (2), Hap-16 (1), Hap-17 (1), Hap-18 (1), Hap-19 (1)
Human	9	9	2	3.500	0.00334	0.389 ± 0.164	Hap-13 (2), Hap-14 (7)

Only a single sequence (from hedgehog) cannot be analyzed for DNA polymorphism

## Discussion

In this study, the overall detection rate of *B. microti* in rodents was 6.64% in central and southern Shanxi, which was lower than that in Yunnan (Lanping: 17.4%, Lianghe: 7.8%) [14], Beijing (12.1%) [26], southeastern Shanxi (10.71%) [19], Henan (9.1%) [28], China, and higher than that in Fujian (3.96%) [29] and Xinjiang (1.8%) [30], China. This variation highlights the regional heterogeneity of *B. microti* distribution and the importance of monitoring in different regions. Among eight rodent species, *B. microti* was detected in *N. confucianus* (16.87%) and *A. agrarius* (3.85%), with no infections observed in the other six rodent species. *Niviventer* *confucianus* is more prone to harboring *B. microti* species, emphasizing the role of *N*. *confucianus* as a key reservoir for *B. microti* in China [31]. However, the limited sample sizes for certain species (e.g., *R. tanezumi*, *n* = 2; *T. triton*, *n* = 9; *M. musculus*, *n* = 8) may bias estimates of host-specific susceptibility. Notably, central Shanxi showed significantly higher detection rates of *B. microti* (9.74%) than southern Shanxi (0.94%); 85.54% of captured *N. confucianus* were from central Shanxi, showing the important role of *N. confucianus* in *Babesia* sp. transmission.

*Babesia microti* is the main causative agent of human babesiosis in China [6, 32]. Previous studies showed that *B. microti* encompassed at least five distinct clades [33]: Clade 1 [*B. microti *sensu stricto (US type)] is a major *Babesia* species causing human babesiosis worldwide [34]; Clade 2 (*Babesia vulpes* type) can infect raccoons, foxes, and badgers [35, 36]; Clade 3 (Munich type) is considered to be nonzoonotic, mainly detected in voles in Europe and North America [37]; Clade 4 (Kobe type) is mainly detected in the rodents in Asia and is pathogenic to humans [13, 38]. Clade 5 (Hobetsu/Otsu types) have been detected primarily in Japan, and no human cases have been reported [38, 39]. Our present study revealed that only *B. microti* Kobe type was prevalent in the rodents in central Shanxi; it has also been detected in many regions of China, such as Yunnan, Zhejiang, Fujian, and Beijing [13, 14, 26]. Phylogenetic analysis confirmed that most sequences from China belong to the *B. microti* Kobe type, which is mainly divided into four clusters; sequences from rodents in Shanxi were in one cluster, sequences from rodents in Yunnan were in one cluster, sequences from rodents in Zhejiang, Fujian, and Beijing were in one cluster, and sequences from humans in Zhejiang and Yunnan were in another cluster, suggesting that the *B. microti* Kobe type is relatively complex, with certain regional and host specificity. This genetic consistency highlights the stability of this genotype in the regional rodent populations.

To further assess the genetic variability of *B. microti*, we performed a comparative analysis of the genetic variability of *18S rRNA* sequences among different hosts in China. Among the 21 haplotypes of *B. microti* observed in China, three distinct haplotypes (Hap-1, Hap-10, and Hap-11) were observed in Shanxi, seven haplotypes (Hap-2 to Hap-8) in Heilongjiang, and 11 haplotypes (Hap-9 and Hap-12–21) in Yunnan, suggesting localized evolutionary diversification of *B. microti*. These haplotypes in Shanxi exhibited close genetic proximity by 1–3 nucleotide substitutions with rodent-derived sequences from Yunnan and Fujian provinces and human-derived sequences from Yunnan and Zhejiang provinces. The predominance of Hap-1 across multiple provinces (Fujian, Yunnan, and Shanxi) implies its ancestral role in the radiation of Chinese *B. microti* lineages, with subsequent host-adaptive mutations generating derivatives, such as Hap-9 (Yunnan rodents) and Hap-14 (Yunnan and Zhejiang human cases). Notably, haplotype network analysis revealed closer genetic linkages between rodent-derived strains from Shanxi and human isolates from Yunnan/Zhejiang, raising concerns regarding zoonotic spillover potential. Many haplotypes (Hap-2 to Hap-8) were observed in tick-derived sequences; however, the genetic similarity among these sequences was very high. The higher genetic diversity observed in tick-derived sequences compared to rodent or human isolates indicated that ticks may act as vectors and evolutionary drivers of *B. microti* diversity. However, the limited haplotype diversity among human cases (Hd = 0.389) may reflect sampling bias or bottlenecks during cross-species transmission.

This study has several limitations. First, uneven sample sizes across rodent species (e.g., 156 *A. agrarius* vs. 2 *R. tanezumi*) may bias estimates of host-specific susceptibility, as low sample sizes reduce statistical power to detect infections in rare species. Second, spatial sampling bias arises from the disproportionate capture of *N. confucianus* in central Shanxi (85.5% of specimens), potentially conflating geographic and host-specific drivers of prevalence. Third, reliance on PCR targeting the *18S rRNA* gene, while effective for screening, may underestimate strain diversity compared to multi-locus sequencing. Future studies should prioritize balanced sampling across species and habitats, integrate vector/human surveillance, and employ high-resolution genotyping to resolve these uncertainties.

## Conclusions

This study represents the first comprehensive investigation integrating genetic data, host ecology, and landscape factors related to *B. microti* in central and southern Shanxi. The high prevalence (9.74%) of zoonotic Kobe-type *B. microti* in central Shanxi rodents indicates elevated infection risk for local residents, particularly during forest and field activities. Haplotype network analysis elucidated the regional evolutionary pattern of Kobe-type strains, tentatively identifying Hap-1 as the progenitor of derivative haplotypes in Shanxi and indicating its potential for cross-regional transmission. These findings offer critical epidemiological insights into *B. microti* in central Shanxi and underscore the need for proactive surveillance implementation to mitigate zoonotic threats.

## Supplementary Information

Supplementary Material 1: Figure S1. Phylogenetic tree of *18S rRNA* gene of *Babesia* species. A: NJ method; B: ME method; C: ML method

Supplementary Material 2: Figure S2. Phylogenetic tree of *18S rRNA* gene of *B. microti*. A: NJ method; B: ME method; C: ML method

Supplementary Material 3: Table S1. List of abbreviations.

## Data Availability

The data supporting the conclusions of this article are included within the article. All genetic sequences generated in this study are deposited in the National Center for Biotechnology Information (NCBI) and available under accession numbers PV076979-PV076998. Please contact the corresponding author if someone wants to request the data from this study.

## Abbreviations

AA: *Apodemus* *agrarius*
AD: *Apodemus* *draco*
CI: Confidence interval
CL: *Cricetulus* *longicaudatus*
EI: *Eothenomys* *inez*
FJ: Fujian Province
HLJ: Heilongjiang Province
ME: Minimum evolution
MM: *Mus* *musculus*
ML: Maximum likelihood
NC: *Niviventer* *confucianus*
NJ: Neighbor joining
OR: Odds ratio
RFF: Returning farmland to forest
RT: *Rattus* *tanezumi*
SX: Shanxi Province
TT: *Tscherskia* *triton*
YN: Yunnan Province
ZJ: Zhejiang Province
