# Supplementary figures and images for: Prevalence and genetic diversity of Babesia microti in rodents from central and southern Shanxi, China
Source: Parasit Vectors. 2025 Jun 22;18:236. doi: 10.1186/s13071-025-06898-6 (PMC12182668; doi:10.1186/s13071-025-06898-6)

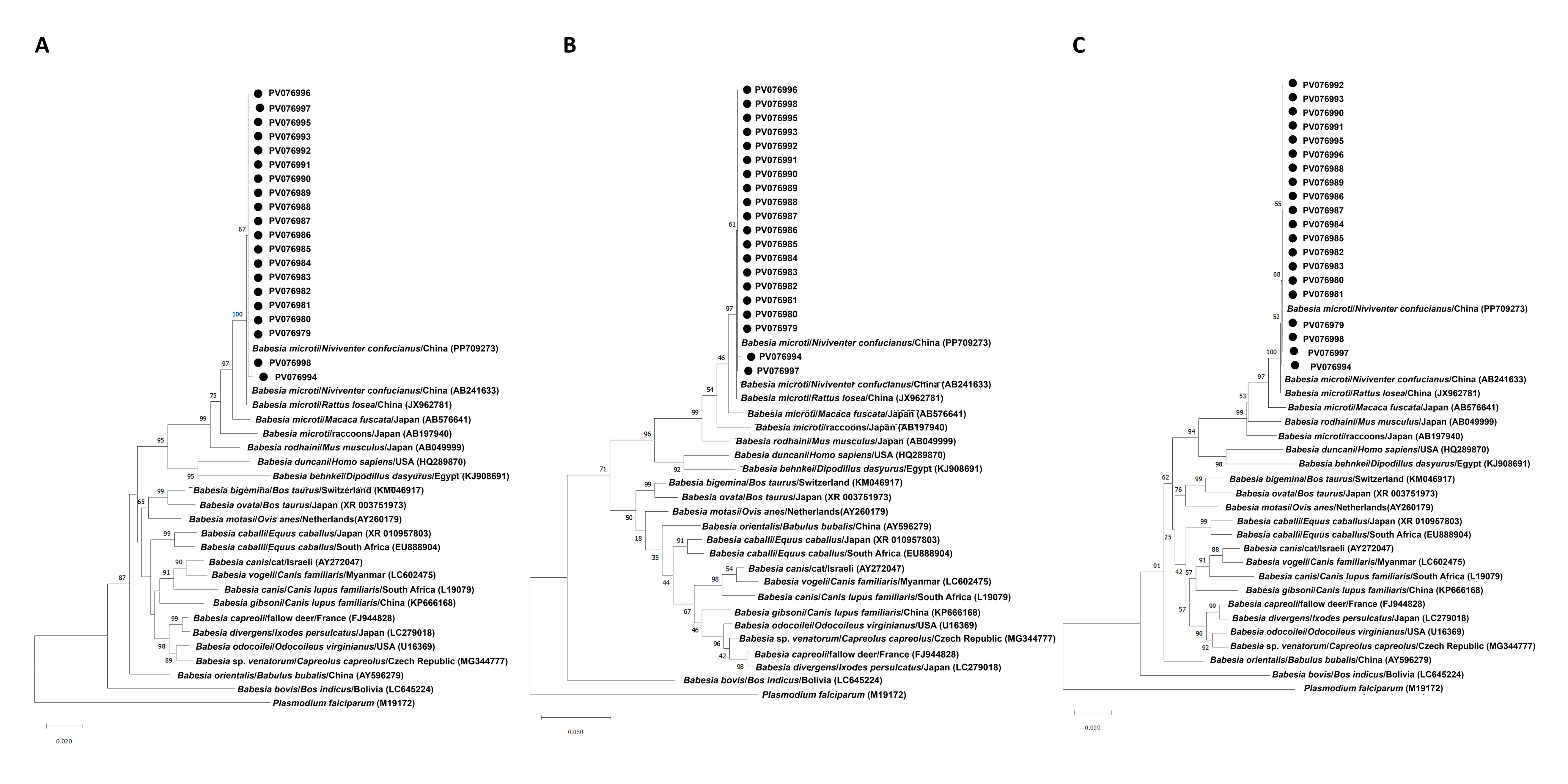

Supplement: Supplementary file 1 — Supplementary Material 1: Figure S1. Phylogenetic tree of 18S rRNA gene of Babesia species. A: NJ method; B: ME method; C: ML method [file 13071_2025_6898_MOESM1_ESM.tif]

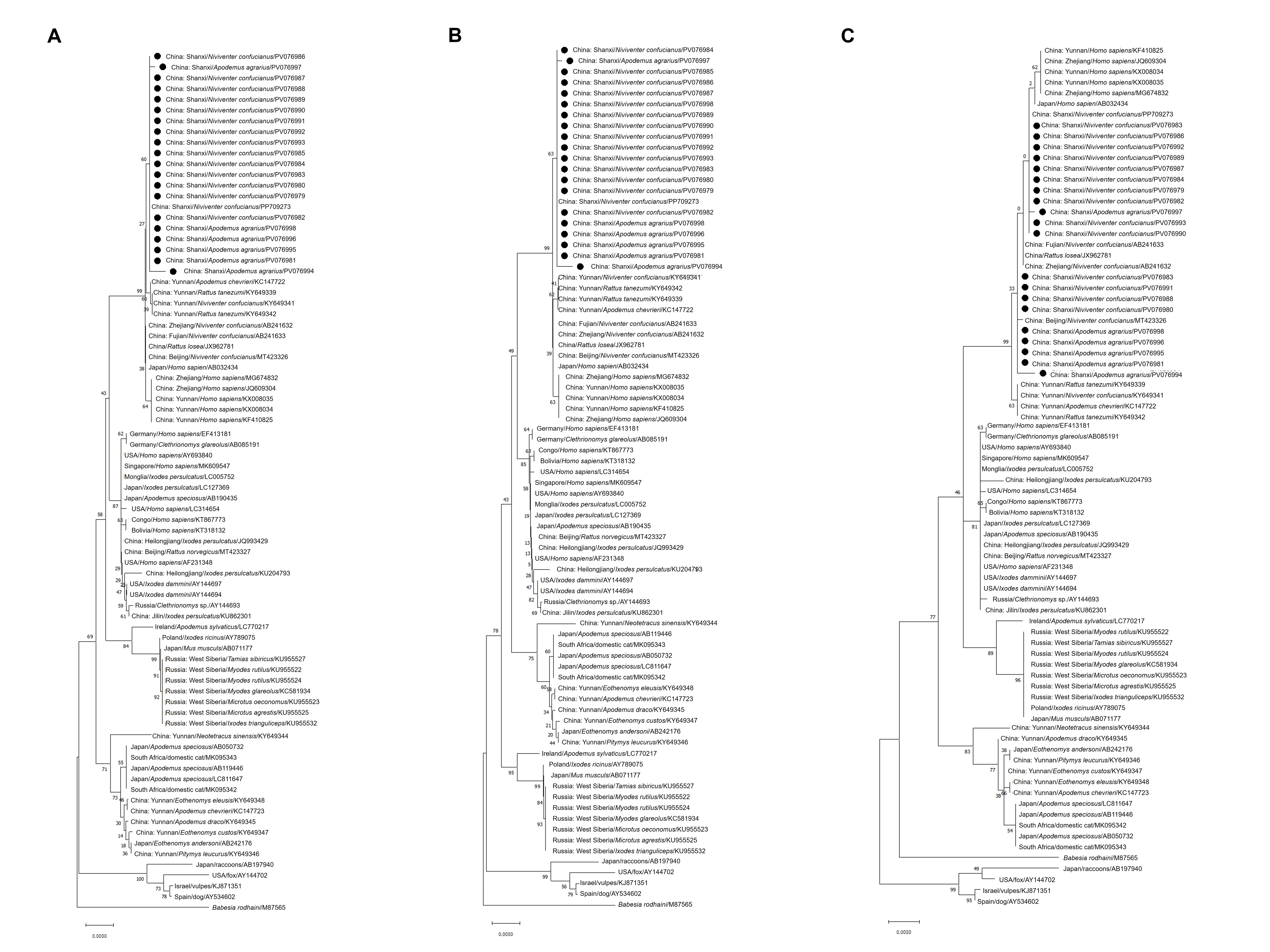

Supplement: Supplementary file 2 — Supplementary Material 2: Figure S2. Phylogenetic tree of 18S rRNA gene of B. microti. A: NJ method; B: ME method; C: ML method [file 13071_2025_6898_MOESM2_ESM.tif]
