# Supplementary material for: Prevalence and genetic diversity of Babesia microti in rodents from central and southern Shanxi, China
Source: Parasit Vectors. 2025 Jun 22;18:236. doi: 10.1186/s13071-025-06898-6 (PMC12182668; doi:10.1186/s13071-025-06898-6)
Supplement: Supplementary file 3 — Supplementary Material 3: Table S1. List of abbreviations. [file 13071_2025_6898_MOESM3_ESM.docx]

Table S1 List of abbreviations

| Abbreviations | Full name |
| --- | --- |
| AA | *Apodemus agrarius* |
| AD | *Apodemus draco* |
| CI | confidence interval |
| CL | *Cricetulus longicaudatus* |
| EI | *Eothenomys inez* |
| FJ | Fujian Province |
| HLJ | Heilongjiang Province |
| ME | minimum-evolution |
| MM | *Mus musculus* |
| ML | maximum likelihood |
| NC | *Niviventer confucianus* |
| NJ | neighbor-joining |
| OR | Odds ratio |
| RFF | returning farmland to forest |
| RT | *Rattus tanezumi* |
| SX | Shanxi Province |
| TT | *Tscherskia triton* |
| YN | Yunnan Province |
| ZJ | Zhejiang Province |
